# Pyrosequencing of Plaque Microflora In Twin Children with Discordant Caries Phenotypes

**DOI:** 10.1371/journal.pone.0141310

**Published:** 2015-11-02

**Authors:** Meng Zhang, Yongxing Chen, Lingzhi Xie, Yuhong Li, Han Jiang, Minquan Du

**Affiliations:** MOST KLOS & KLOBM, School & Hospital of Stomatology, Wuhan University, Luoyu Road 237, Wuhan City, Hubei, China; University Hospital of the Albert-Ludwigs-University Freiburg, GERMANY

## Abstract

Despite recent successes in the control of dental caries, the mechanism of caries development remains unclear. To investigate the causes of dental decay, especially in early childhood caries, the supragingival microflora composition of 20 twins with discordant caries phenotypes were analyzed using high-throughput pyrosequencing. In addition, the parents completed a lifestyle questionnaire. A total of 228,789 sequencing reads revealed 10 phyla, 84 genera, and 155 species of microflora, the relative abundances of these strains varied dramatically among the children, Comparative analysis between groups revealed that *Veillonella*, *Corynebacterium* and *Actinomyces* were presumed to be caries-related genera, *Fusobacterium*, *Kingella* and *Leptotrichia* were presumed to be healthy-related genus, yet this six genera were not statistically significant (P>0.05). Moreover, a cluster analysis revealed that the microbial composition of samples in the same group was often dissimilar but that the microbial composition observed in twins was usually similar. Although the genetic and environmental factors that strongly influence the microbial composition of dental caries remains unknown, we speculate that genetic factors primarily influence the individual's susceptibility to dental caries and that environmental factors primarily regulate the microbial composition of the dental plaque and the progression to caries. By using improved twins models and increased sample sizes, our study can be extended to analyze the specific genetic and environmental factors that affect the development of caries.

## Introduction

There are many methods for the prevention and treatment of caries, especially early childhood caries (ECC). Studies have shown how to alleviate the pain of children's dental caries[1] and have revealed its impact on the development of the permanent teeth. However, this complex, chronic, multi-factorial disease[2] is still common and affects nearly two-thirds (66%) of children in China[3].

Researchers continue to explore the etiology and pathogenesis of caries. In the 1960s, dental decay was suggested to be the result of the interaction of four major factors: biofilm, diet, host and time[4]. Since then, considerable attention has been given to the genetic and non-genetic factors that influence caries development, such as environmental factors, the individual’s health, behavioral habits, lifestyle, dietary composition, and access to dental care[5]. However, due to the complex nature of dental decay and to the limitations in the research approaches, the exact mechanisms of caries development remain unclear.

With recent advances in molecular biology, researchers are now elucidating the structure and function of the oral microbial community and are exploring the relationships between microbial diversity and oral health and disease in different populations. Such in-depth studies of microbiota can pave the way for a greater understanding of the etiology of disease. In recent years, pyrosequencing and phylogenetic analysis of the 16S rRNA gene have proved to be more sensitive and discriminatory compared with traditional PCR cloning because they avoid the biases that are inherent in cloning methods[6, 7]. 16S rRNA pyrosequencing has been used to quantitate the relative abundances of bacterial components and the structural variations of communities[8–10]. Keijser et al[11] examined the bacterial diversity in the healthy adult oral cavity using pyrosequencing and found that the bacterial phylotypes were more complex than previously reported.

Moreover, due to the particular genetics of twins[12], various twins models were used to explore how genetic and environment factors influence several oral cavity and physical diseases. Dicksved[13] sampled the fecal microbiota from both monozygotic twin pairs with Crohn’s disease (discordant and concordant) and healthy twin pairs to study the effects of genetic and environmental factors on the microbial composition of the gut. Turnbaugh[14] attempted to analyze how genetic, environment and adiposity factors influence the gut microbiome by characterizing the fecal microbiota of monozygotic (MZ) and dizygotic (DZ) twin pairs who were concordant for obesity or leanness as well as their mothers. Corby[15] uncovered several microbial risk indicators of ECC by studying the samples of 204 twin pairs. However, pyrosequencing analysis of dental caries using twin pairs with discordant phenotypes was rarely been reported.

Therefore, to explore ECC further, we used pyrosequencing to determine the composition of the supragingival plaque microbiota in 10 pairs of twin children with discordant caries phenotypes. We then used a questionnaire survey to speculate on the factors that resulted in the different caries phenotypes in the twins.

## Materials and Method

### Ethics Statement

Informed written consent was obtained from the parents of all participants prior to enrollment in this study. The study design, protocol, and informed consent form were approved by the Ethics Committee at the School & Hospital of Stomatology, Wuhan University (Wuhan, China).

### Subject Selection

The twins, aged 3–6 years, came from four different kindergartens in Wuhan, China. In each twin pair, only one child had caries and all individuals were primary dentition. The definition and diagnosis of dental caries were based on the criteria of the International Caries Detection and Assessment System (ICDAS, 2005)[16]. The “Caries” group subjects had more than 3 cavitated caries teeth which includes at least one carious primary molar(codes 5 or 6 based on the classification of the carious status of the ICDAS), whereas the “Healthy” group subjects did not have caries in any teeth (code 0 based on the classification of the carious of the ICDAS). All subjects were examined clinically by a single calibrated examiner before sampling, and the samples were subsequently divided into three groups: Hhc1 group (H1: intact enamel surface of healthy individuals, n = 10); Hhc2 group (H2: intact enamel surface of caries individuals, n = 10); and Chc2 group (C2: decayed tooth surface of caries individuals, n = 10).

The inclusion criteria were that the children were medically healthy, had no current bacterial or viral infections, had not used antibiotics within the preceding three months, had more than 18 teeth present, had no enamel or dentin hypoplasia detectable visually, and had not received any prior treatment (including fluoride) for dental caries. In addition, the parents filled out a questionnaire mainly covers the following four aspects, 1) basic personal information.(includes the zygotic of twins) 2) daily habits. caregivers, life habits, oral health awareness and oral status of parents and caregivers. 3) oral health behavior. feeding within three months, brushing start time, brushing frequency and time of day, brushing assistance. 4) eating habits. dietary preferences, poor eating habits, mainly concern on caries-related dietary factors.

### Sample Collection and Preparation

Samples were collected in the morning, and the children were asked not to brush their teeth before the exam. The plaque samples of the non-caries children were collected from at least three healthy surfaces, including anterior and posterior teeth, and were then pooled. The plaque samples of the caries-active subjects were collected separately from two types of surfaces: the surface of intact enamel (site 1) and the surface of a deep caries lesion (site 2). All plaques were sampled 2 h after breakfast and were obtained after rinsing by scraping the tooth surface with a sterile curette. The curette was immersed in a sterile 1.5 ml Eppendorf tube that contained 1 ml of a saline solution that had been UV-irradiated to eliminate DNA contamination. The samples were transported on ice to the laboratory and stored at −80°C unt0il used.

### Taxonomic Assignment of Individual Sequencing Reads

The detail of total genomic DNA extraction and amplification of 16S rRNA genes are described in the Supporting Information(S1 File).

### Sequencing Data and Statistical Analysis

We designed three different grouping ways and conducted comparative analysis respectively. 1) Grouped according to the health condition of the teeth (H1, H2, C2). The comparative analysis within and among the groups were been conducted. 2) Grouped according to the twin pairs(a total of 10 groups). We only conducted comparative analysis between samples within the group. 3) Grouped according to the kindergarten(K1, K2, K3, K4). The comparative analysis within and among the kindergartens were all been conducted. The details of sequencing data and statistical analysis are described in the Supporting Information(S1 File).

### Accession Numbers

All sequences and associated metadata were deposited into the NCBI Sequence Read Archive under the accession number SRP062988.

## Results

### Subjects and Samples

A total of 30 supragingival samples (T1-T30) were sampled from 10 pairs of twins aged 3–6 years (10 males and 10 females, Table 1). Samples T1-T6, T7-T12, T13-T21, and T22-T30 were from different kindergartens (K1, K2, K3, K4). In addition, the parents of each pair of twins filled out a lifestyle questionnaire.

**Table 1:** Sample Information and Pyrosequencing Data.

Subject/Sample name		Group	Sex	Raw reads number	Effective reads number	OTU number
K1-1^a^	T1	Hhc1	F	10687	7904	142
K1-1^b^	T2	Hhc2	M	13474	9110	146
	T3	Chc2		7868	5921	127
K1-2^a^	T4	Hhc1	M	9089	6653	170
K1-2^b^	T5	Hhc2	F	9271	6785	160
	T6	Chc2		14070	10656	133
K2-1^a^	T7	Hhc1	M	17059	12919	238
K2-1^b^	T8	Hhc2	F	20196	15018	261
	T9	Chc2		17942	13373	242
K2-2^a^	T10	Hhc1	M	14302	10806	182
K2-2^b^	T11	Hhc2	F	12801	9225	200
	T12	Chc2		13153	9486	202
K3-1^a^	T13	Hhc1	F	10145	7609	161
K3-1^b^	T14	Hhc2	F	9059	6746	122
	T15	Chc2		16852	12378	210
K3-2^a^	T16	Hhc1	M	12109	8887	159
K3-2^b^	T17	Hhc2	M	13213	9824	162
	T18	Chc2		12254	9058	166
K3-3^a^	T19	Hhc1	F	14595	10844	198
K3-3^b^	T20	Hhc2	F	11735	8708	173
	T21	Chc2		14706	10656	224
K4-1^a^	T22	Hhc1	M	4482	2851	100
K4-1^b^	T23	Hhc2	M	3923	2553	86
	T24	Chc2		4030	2709	67
K4-2^a^	T25	Hhc1	F	4497	2784	81
K4-2^b^	T26	Hhc2	F	5198	3486	75
	T27	Chc2		5870	3737	71
K4-3^a^	T28	Hhc1	M	4262	2891	69
K4-3^b^	T29	Hhc2	M	3890	2597	71
	T30	Chc2		4273	2615	68

K1, K2, K3, K4 denote four different kindergartens;

-n(n = 1, 2, 3) represent one of the twin pairs in this kindergarten;

M = male, F = female.

^a^ The healthy individuals

^b^ The caries individuals

### Pyrosequencing Data

Pyrosequencing of the 16S rRNA gene amplicons yielded 315,005 high-quality reads, of which 228,789 reads (72.6%) passed quality control. There were 152,025 unique sequences, and they represented all phylotypes. The average length of the sequences was 397 bp, excluding the primers. The number of sequences per sample ranged from 2553 to 15018 (mean read = 7626) (Table 1).

The operational taxonomical unit (OTU) diversity was assessed using the program Mothur. Overall, 1,967 OTUs were detected, with each sample averaging 656 OTUs. S1 Fig shows the degrees of OTU sharing and non-sharing among the three groups. After the duplicate sequences were removed, the three groups shared 169 OTUs. Mothur identified a higher number of OTUs for the C2 group (mean OTU = 881) compared with the H1 and H2 groups (mean OTU = 820 and 804, respectively), but the comparisons of the alpha diversity index (S2 Fig) indicated that the differences among the three groups were insignificant (P>0.05. The rarefaction curves showed the curves did not reach the saturation level, which suggests that the microbial richness of the supragingival plaque was not completely revealed at the current sequencing depth.

**Fig 1:**
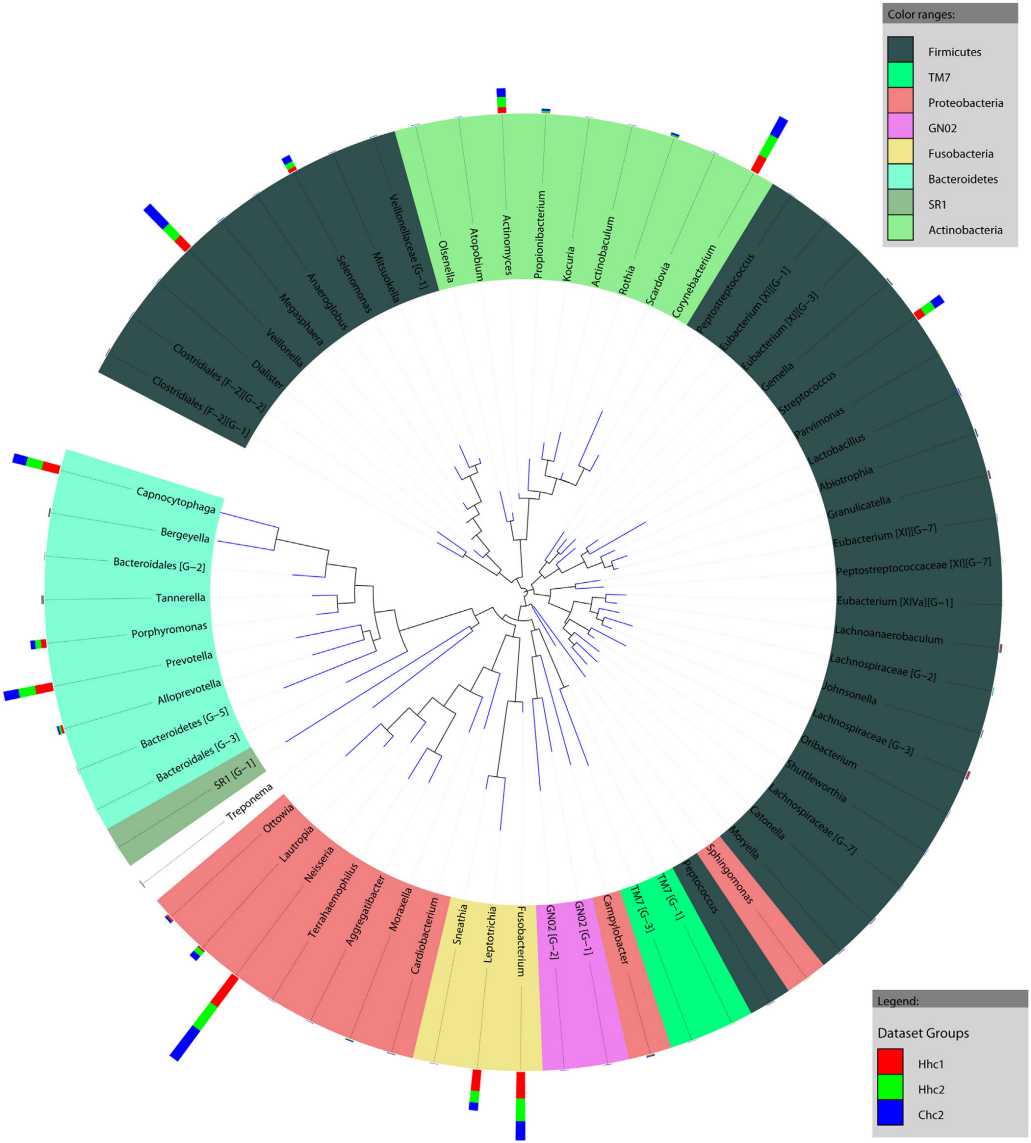
Circular maximum likelihood phylogenetic tree at level of genus. The inner band shows genera colored by their corresponding phylum (see key for taxa with multiple members), the outer band shows overall relative abundance of each genus in different groups. The tree was constructed in iTOL (Letunic and Bork, 2007).

Fig 1 shows the phylogenetic tree at the level of genus. The overall abundance and magnitude of the differences among the three groups are indicated by bars.

### Composition and Distribution of Supragingival Microbiota

**Fig 2:**
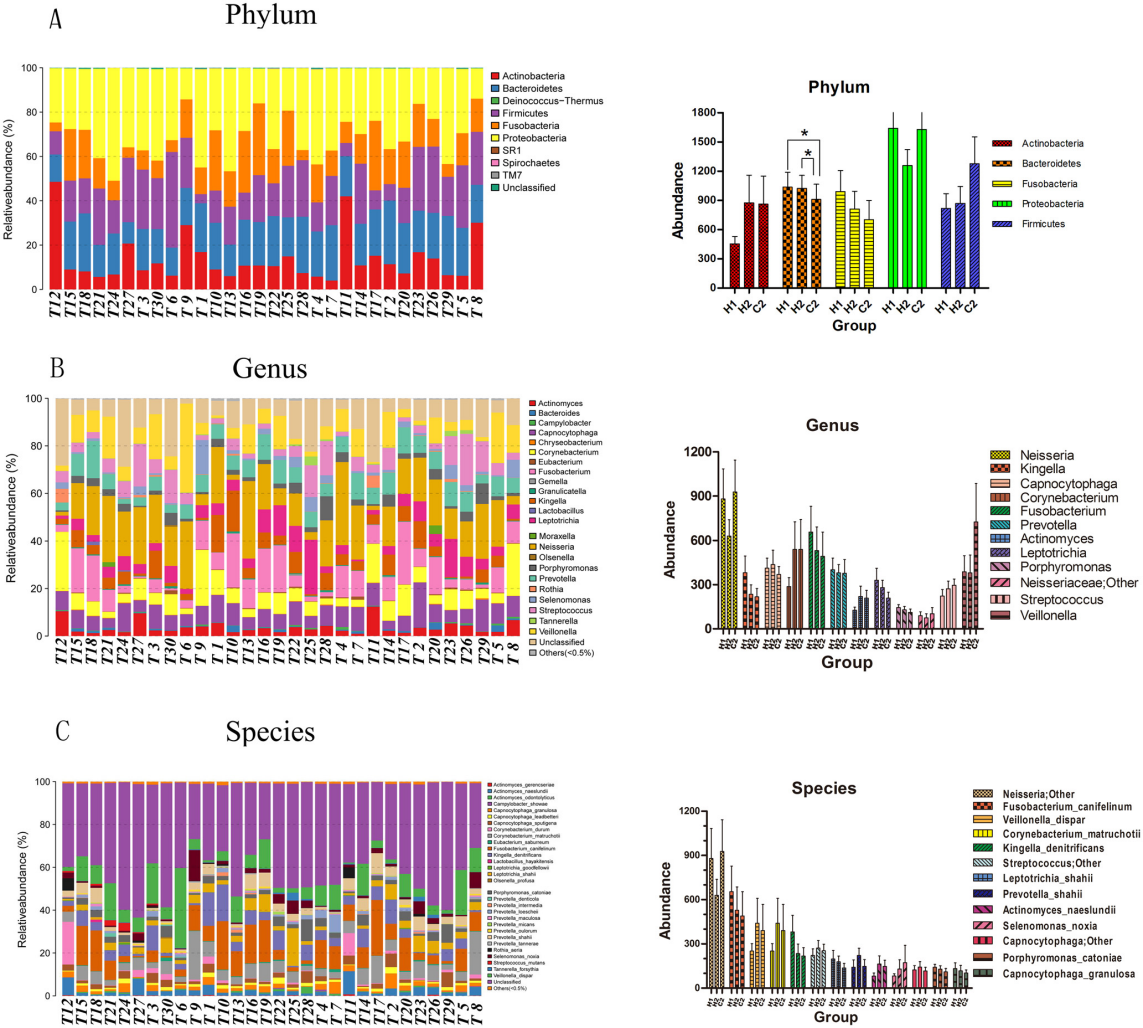
The barplot graph of samples microorganisms and the predominant bacteria of three groups. (A, B, C) Abundance and prevalence of bacteria at the phylum, genus, and species level in the 30 plaque samples. (a, b, c) Mean levels of the predominant bacteria in groups H1, H2 and C2 at the phylum, genus, and species level.

In a BLAST search against the HOMD, a total of 10 phyla, 20 classes, 33 orders, 55 families, 84 genera, and 155 species were detected. The proportions of the predominant taxa at the phylum, genus, and species levels were compared among the groups (Fig 2). The classification of sequences by phylum is summarized in Fig 2A. The vast majority of sequences belonged to these five phyla: Proteobacteria (29.8%), Bacteroidetes (19.5%), Firmicutes (19.5%), Fusobacteria (16.5%), and Actinobacteria (14.4%). In group C2, Firmicutes and Actinobacteria were found at relatively high abundance (P>0.05), whereas Fusobacteria and Bacteroidetes were found less frequently. Among the five common phyla, only Bacteroidetes had a significant difference between the caries-free plaques (H1 and H2 group) and caries-active plaques (C2 group) (P = 0.0370 and P = 0.0220, respectively). The TM7 phylum was found only in groups H1 and H2, the DSR1 phylum was found only in group H2, and the Deinococcus-Thermus phylum was found only in group C2.

At the genus level (Fig 2B), a total of 84 genera were represented, with 50 genera shared by the three groups. Approximately 87% of the sequences came from twelve genera: *Neisseria*, *Fusobacterium*, *Veillonella*, *Corynebacterium*, *Capnocytophaga*, *Prevotella*, *Kingella*, *Leptotrichia*, *Streptococcus*, *Actinomyces*, *Porphyromonas*, and *Selenomonas*. In comparing these genera, we found that *Veillonella* (48.6% vs 26%), *Corynebacterium* (39.8% vs 21%), and *Actinomyces* (37.6% vs 22.9%) were found more frequently in group C2 than in group H1. In contrast, *Fusobacterium* (29.3% vs 39.1%), *Kingella* (26.1% vs 45.7%), and *Leptotrichia* (25.5% vs 40.3%) were found more frequently in group H1 than in group C2. However, the differences in representation of the above six genera were not statistically significant (P>0.05). The genera that did exhibit significant differences in representation between groups C2 and H1 were *Lactobacillus* (P = 0.0380), *Propionibacterium* (P = 0.0010), *Scardovia* (P = 0.0321), *Moraxella* (P = 0.0010), *Alysiella* (P = 0.0010), and *TM7-genus-incertae-sedis* (P = 0.0028), although these were found infrequently (0.004–0.21%). Approximately 10% of all sequences could not be identified at the genus level.

At the species level (Fig 2C), thirteen species constituted 73.4% of the oral microbiota, and 5 species (*Neisseria;Other*, *Fusobacterium_canifelinum*, *Veillonella_dispar*, *Corynebacterium_matruchotii*, and *Kingella_denitrificans*) accounted for 49.3% of the total. An unclassified *Neisseria* species (12.61%-34.85%) was the most abundant strain in many of the samples. In addition, we found a relatively high level of *Veillonella_dispar* (13.2%), *Corynebacterium_matruchotii* (7.2%), and *Selenomonas_noxia* (3.2%) in group C2 as well as *Fusobacterium_canifelinum* (13.2%) and *Kingella_denitrificans* (7.7%) in group H1. However, the differences in representation of these species among the groups were not statistically significant; only low-abundance species had significant differences in representation. Furthermore, the abundance of most major genera and species in group H2 was similar to their abundance in groups H1 or C2 (Fig 2B and 2C).

### Comparative analysis of the differences within and between the twins

**Fig 3:**
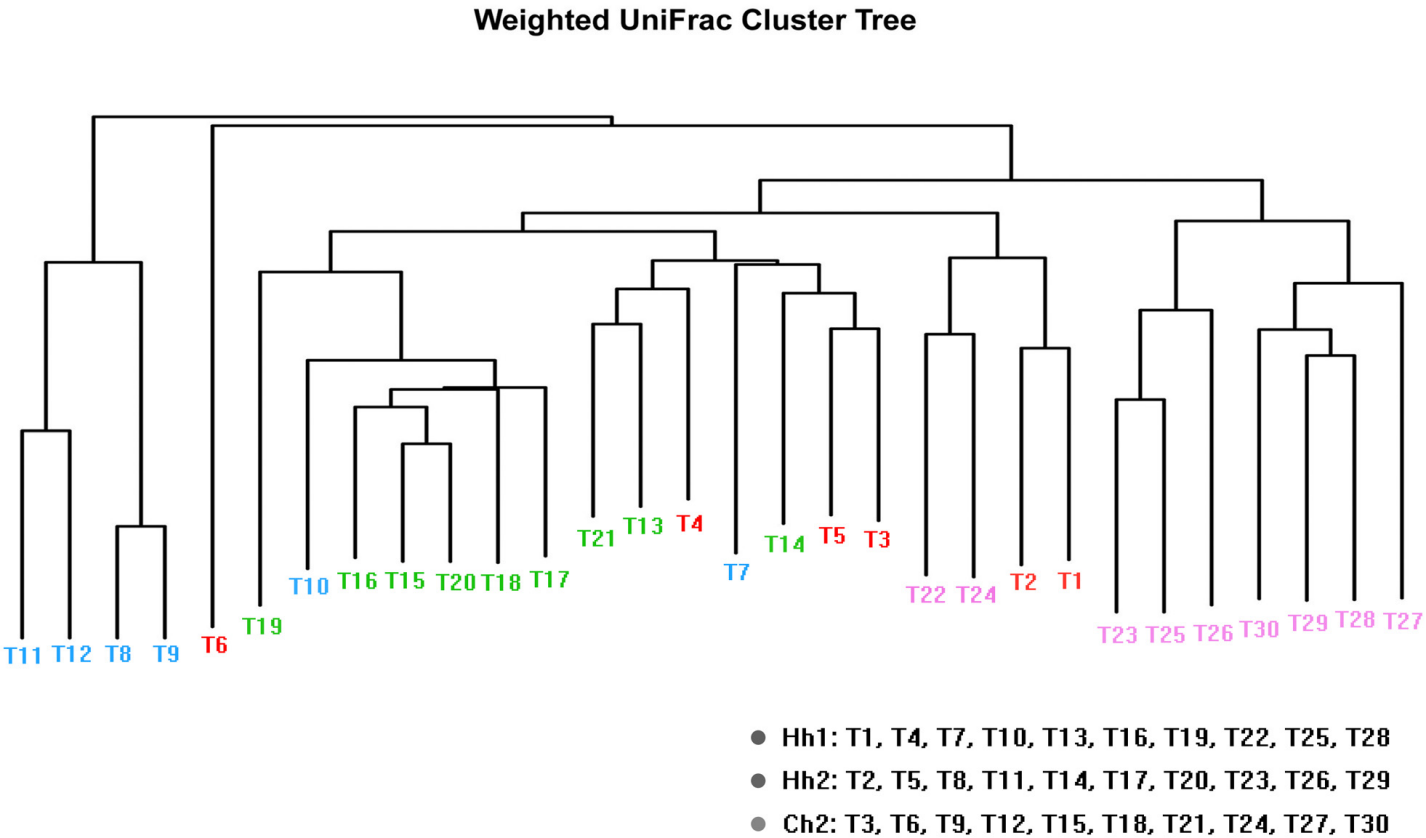
Weighted Unifrac clustering results of the samples. Samples having similar plaque microbiota compositions are usually clustered in a sub-branch. The different colors denote different kindergartens.

**Fig 4:**
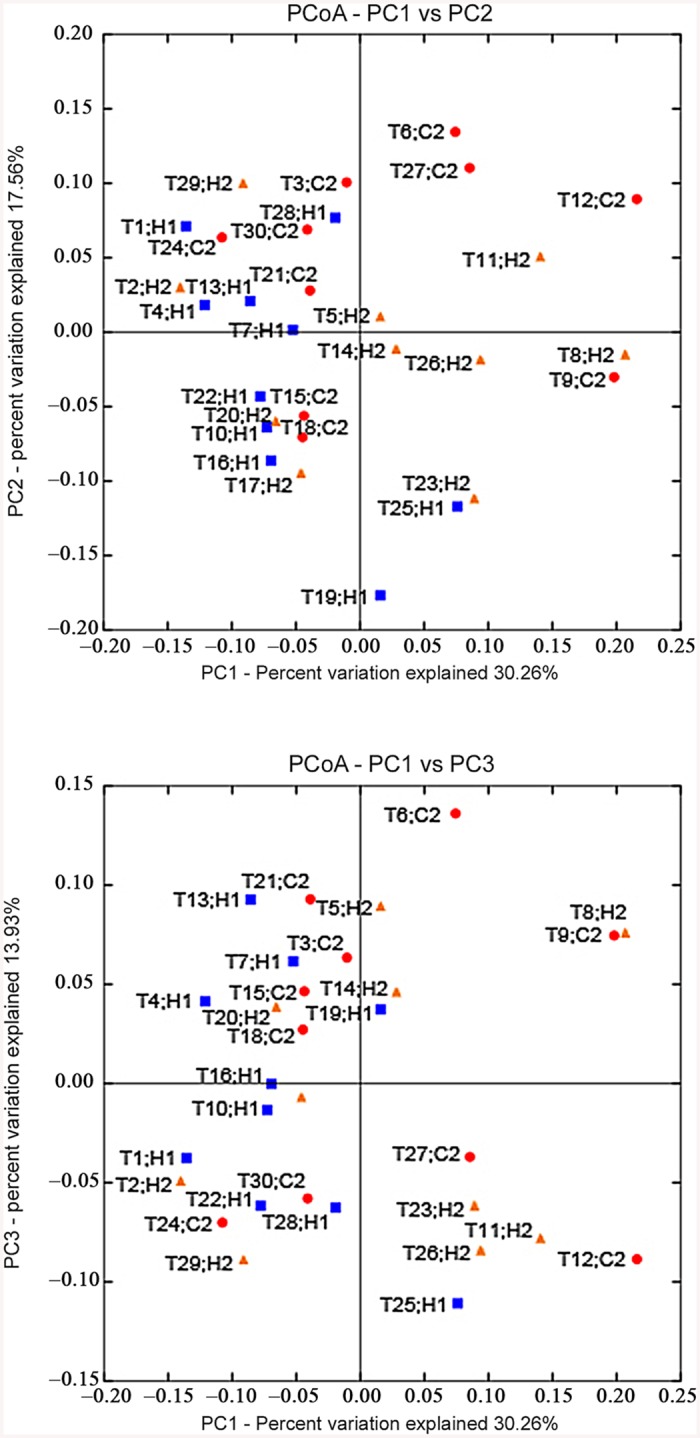
Principal Component Analysis results on individual samples. Principal Component Analysis (PCA) results on all individual samples at the level of OTUs clustering sequences at a 3% difference: A) the plot of the PCA axis 1 (accounting for 30.26% of intersample variation) and the axis 2 (17.56% of intersample variation); B) the plot of the PCA axis 1 and the axis 3 (13.93% of intersample variation). Blue dots—samples(Square) from group H1, orange dots—samples(Triangle) from groupH2, red dots—samples(Round) from group C2. Data were normalized to an equal number of reads per sample and log2 transformed.

According to a cluster analysis (Fig 3), samples that are clustered in a sub-branch have similar plaque microbiota compositions. In the present study, the samples of a group generally did not cluster in one branch: the samples of group H2 clustered with samples of group H1 or group C2 (in addition to T5, T14, T20). A principal component analysis(PCoA) (Fig 4) showed a similar tendency in the distances based on genus level.

In addition, the results of the questionnaire showed that most of the twin pairs share the similar daily habits, oral health habits and eating habits. Only a few twins(T4-T6, T13-T15, T19-T21) have some differences in dietary preferences. When the sequencing data were combined with the questionnaire data (S1 Table), we found that the clustered samples, (Fig 3) such as T1-T6, T7-T12, T13- T21, and T22-T30 (except for some special cases), coincides with the 4 different kindergartens. This finding indicates that twin children living in the same environment have relatively similar plaque microflora. The plaque composition of different twin pairs in the same kindergarten had some cross-similarity, which indicates that that even with different genetic backgrounds, the environment affects the composition of plaque to a certain extent. In addition, the three samples of each twin pair usually clustered together as T1-T3, T7-T9, T10-T12, T16-T18, T22-T24, T25-T27, and T28-T30. This likely occurred because the twins have at least 50% identical genes and share similar living conditions, oral health behavior and daily habits. When the two individuals of a twin pair differed in their diet preferences (caries-related factors: eat sweets or snacks, drink carbonated beverages), their samples were farther apart, such as T4-T6, T13-T15, and T19-T21. This finding indicates that caries-related dietary differences also influence plaque composition.

**Fig 5:**
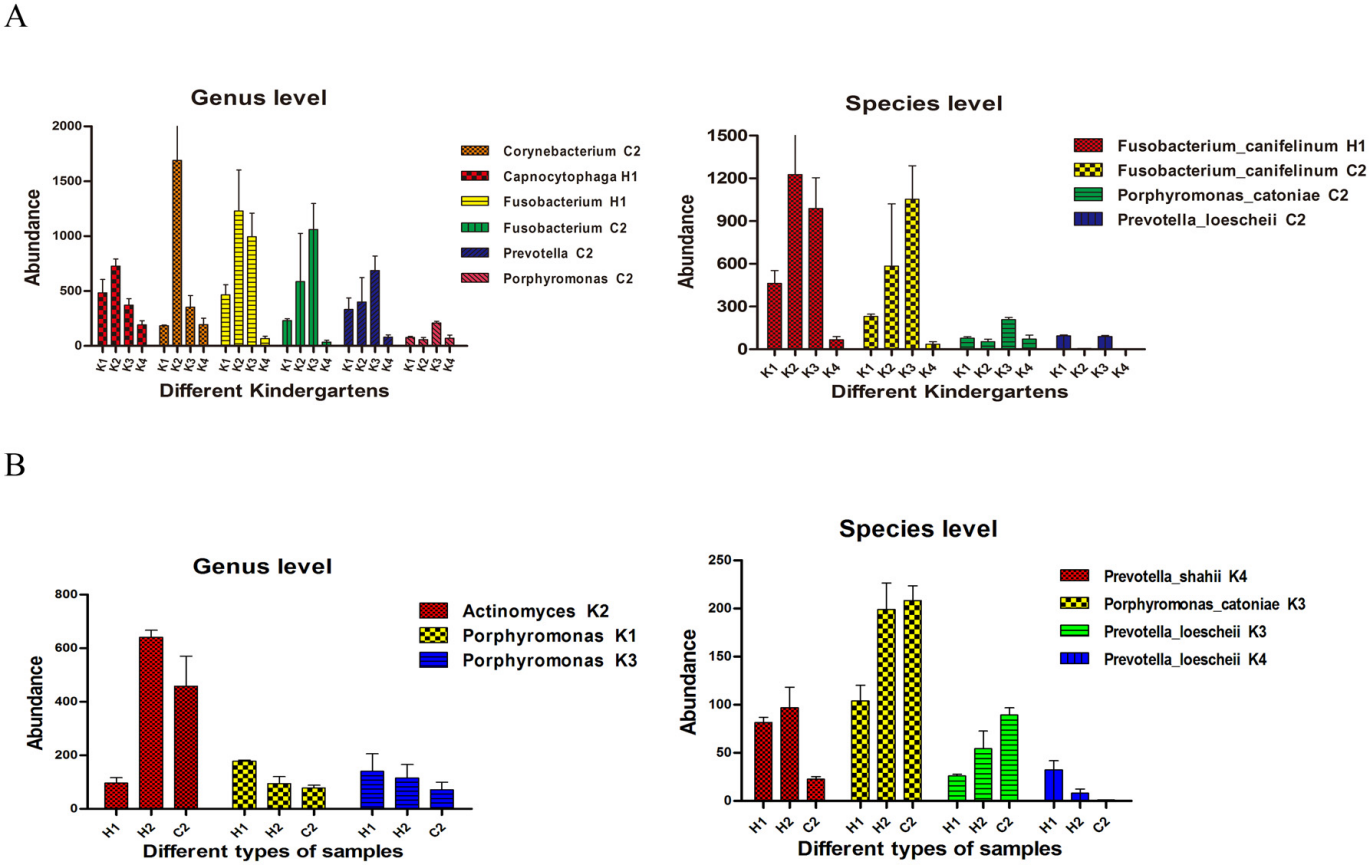
Genera and species with significant differences in representation (P<0.05). A: Comparisons between different kindergartens. B: Comparisons between different samples in the same group.

Moreover, the same type of samples in different kindergartens and the different types of samples in the same kindergarten were analyzed. The bacteria that exhibited significant differences in this analysis are shown in Fig 5. The comparison of group C2 among different kindergartens was relatively prone to differences. In contrast, differences were rarely found when comparing samples in the same kindergarten.

## Discussion

With the completion of the Human Genome Project and the recent advances in molecular biology, innovative molecular applications of various twins models have the potential to advance our understanding of the etiologies of complex diseases and disorders. Dental caries is a multi-factorial, polygenetic, unintelligible oral disease with an unclear pathogenesis. Therefore, twins with discordant caries phenotype were selected in this study to explore the etiology of dental decay. However, after screening nearly 80 pairs of twins, only 10 pairs met our requirements, and eight of those ten (80%) were dizygotic twins (the other two were unknown). This provides further evidence that monozygotic twins have a more similar caries experience but that different-sex dizygotic twins have the greatest variance[17]. Unfortunately, this selection greatly reduced our sample size.

Because the mouth is in continuous contact with the external environment, the oral microbiome is extremely dynamic[18]. Among the oral microbiota, the supragingival plaque may have the most impact on the incidence of dental caries in the micro-environment[19–20]. However, most previous studies on dental plaque only collected carious teeth plaque from caries individuals [15, 21]. In our study, the intact enamel surface plaques of caries individuals were also sampled. This may help us further comprehend the plaque microbiome community.

To effectively analyze plaque microbiota structure and function, different hypervariable regions (V1-V9) should be selected carefully for accuracy and reliability. Claesson et al[22] constructed a library of human fecal microbiota using the V3/V4 and V4/V5 regions because these microbiota were predicted to provide the highest accuracies for phylogenetic assignment. Therefore, in our study, the V3-V5 hypervariable regions were amplified and sequenced for analysis (Escherichia coli positions 517F-907R).

Studies[23] have shown that bacterial diversity decreases significantly with the aggravation of caries; however, the differences in bacterial diversity among the three groups was not significant in our study[24, 25]. This may be due to the subjects whom we chose. A study reported that the number of shared species and the similarity of the plaque microbiota between twins are great[26]. Thus, although the twins included in our study were discordant for caries, their plaque compositions were similar. In this case, after increasing the sample size, the differences in microbiota among the different groups would still be very small.

In our study, approximately 70% of the microbiota were represented by five phyla, twelve genera and thirteen species. Wen et al[27] reported that these five major phyla have no significant differences in representation among children. In another report[28], Firmicutes was shown to be the prevailing phylum in supragingival plaques of severe ECC patients. Wen[29] and Belda-Ferre[30] reported that Bacteroidetes was represented to a greater extent in caries-active samples than in caries-free samples; however, in our study, Bacteroidetes (P<0.05) was represented to a lesser extent in caries-active samples (group C2). This inconsistency might be caused by the different primer sets that selected different hypervariable regions of the 16S rRNA gene, by different degrees of caries of the subjects, or both. At the genus level, previous studies have indicated that the cariogenic bacteria *Streptococcus*, *Veillonella*, *Actinomyces*, and *Leptotrichia* accounted for a large proportion of the microbiota in caries subjects[31, 32]. Our study showed that *Leptotrichia* may be associated with healthy teeth[33]. In addition, *Abiotrophia* has been reported to account for a significant proportion of the microbiota on the healthy enamel surface in both children and adults[9], but we did not detect this bacteria. At the species level, more phylotypes were detected in our study than were found in previous studies[11]. However, most of the bacteria were represented by only 13 species. Though the relative abundances of these major bacteria varied dramatically between samples, significant differences were rarely detected, which may be have relation with our subjects.

Although each twin pair was discordant for the caries phenotype, a cluster analysis indicated that the twins’ plaque compositions were similar. Furthermore, large differences in the representation of several predominant phylotypes were detected in different kindergartens. In the current study on dental caries, we thus speculate that phenotypic differences likely arose because the quality and/or quantity of several specific cariogenic strains on the surface of healthy teeth did not match the cariogenic conditions, although the plaque compositions of caries-active and caries-free teeth were similar. Compared with the teeth of unrelated healthy subjects, group H1/H2 may be more susceptible to caries. This finding indicates that an increase or decrease of any one phylotype does not necessarily lead to the occurrence of dental caries; the appropriate microbiological composition and the combined effects of the cariogenic bacteria causes dental caries. This view is consistent with the ‘Ecological Plaque Hypothesis’[34].

Evidence from animal models[35] and heritability estimates of different twins models[36] indicates that a significant proportion of the factors that influence the incidence and severity of caries is heritable, such as the structure of dental enamel[24], immunologic response to cariogenic bacteria[37], sugar metabolism[38] and the saliva constituents[39], which in turn indicates that elements of the host genome are significant risk factors in the etiology of dental caries. In addition, these studies demonstrated that genetic factors significantly influence host taste preferences[40], whereas environmental factors primarily influence microbial acid production[41]. Unfortunately, our study did not yield definitive answers regarding the relative influences of genetic and environmental factors on the development of dental caries. However, the combination of our study results and those of recent studies in dental decay suggest that genetic factors primarily influence an individual's susceptibility to dental caries but that environmental factors primarily regulate the composition of the dental plaque and the progression to caries. More in-depth studies with larger groups sizes and more diverse samples are needed to validate this hypothesis.

Studies on MZ twin pairs[26] who show different phenotypes for a particular trait or disease provide a powerful approach for clarifying the roles of genetic and environmental influences on normal features or susceptibility to diseases. However, it may be difficult to collect a sufficient number of suitable samples. In the present study, because the data between the groups were not significantly different, correlation analyses and model predictions would have been unreliable[25, 42]. In future studies, we will select MZ twins with discordant phenotypes and increase the group sizes to further elucidate the specific genetic and environmental factors that affect the development and progression of caries.

## Supporting Information

S1 Fig Venn diagram of the number of OTUs that are shared unique among the groups (Hhc1, Hhc2, Chc2). The interior of each large circle symbolically represents the number of OTUs found in the group. The overlapping areas or intersections represent the number of OTUs found in both (or all three) groups. The single-layer zone represents the number of OTUs found only in that group.
(TIF)

S2 Fig Comparison of phylotype coverage and diversity estimation at 3% dissimilarity from the pyrosequencing analysis. P values of comparisons of three groups were shown in the last table. The coverage percentage (Observed-species), richness estimators (Chao and ACE), diversity indices (Shannon and Simpson) were calculated using the Mothur program.
(TIF)

S1 File The appendix of “Pyrosequencing of Plaque Microflora In Twin Children with Discordant Caries Phenotypes.” (DOC)

S1 Table Summary of questionnaire. Daily habits including caregivers, life habits, oral health awareness and oral status of parents and caregivers.Oral health habits including feeding within three months, brushing start time, brushing frequency and time ofday, brushing assistance.Eating habits including dietary preferences, poor eating habits, mainly concern on caries-related dietary factors.
(DOC)
