# Supplementary figures and images for: Pyrosequencing of Plaque Microflora In Twin Children with Discordant Caries Phenotypes
Source: PLoS One. 2015 Nov 2;10(11):e0141310. doi: 10.1371/journal.pone.0141310 (PMC4629883; doi:10.1371/journal.pone.0141310)

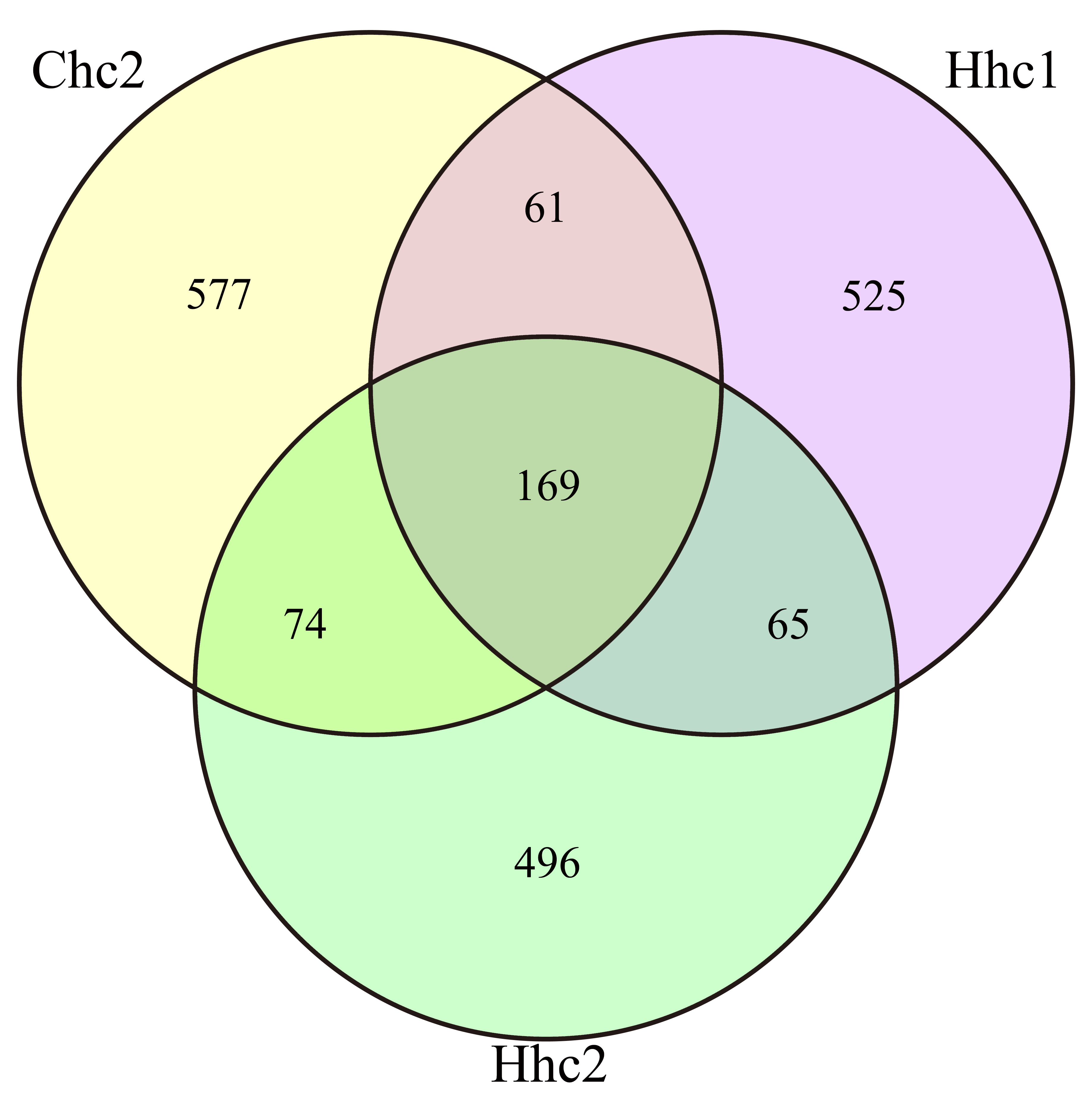

Supplement: S1 Fig — The interior of each large circle symbolically represents the number of OTUs found in the group. The overlapping areas or intersections represent the number of OTUs found in both (or all three) groups. The single-layer zone represents the number of OTUs found only in that group. (TIF) [file pone.0141310.s001.tif]
