# Supplementary material for: Pyrosequencing of Plaque Microflora In Twin Children with Discordant Caries Phenotypes
Source: PLoS One. 2015 Nov 2;10(11):e0141310. doi: 10.1371/journal.pone.0141310 (PMC4629883; doi:10.1371/journal.pone.0141310)
Supplement: S1 File — (DOC) [file pone.0141310.s003.doc]

# Pyrosequencing of Plaque Microflora In Twin Children with Discordant Caries Phenotypes

Meng Zhang^1^, Yongxing Chen^1^, Lingzhi Xie^1^, Yuhong Li^1^, Han Jiang^1^, Minquan Du^1*^

^1^ MOST KLOS & KLOBM, School & Hospital of Stomatology, Wuhan University, Luoyu Road 237, Wuhan City, Hubei, China.

* corresponding author

E-mail: [duminquan@whu.edu.cn](mailto:duminquan@whu.edu.cn)

**Total Bacterial Genomic DNA Extraction**

Bacteria in the plaque samples were collected by centrifugation at 13,200×g for 2 min. The bacteria were suspended in 480 µl of EDTA, 120 µl of lysozyme buffer and 2 µl of Mutanolysin and were then incubated at 37 °C for 60 min. The bacteria were centrifuged again and then suspended in lysis buffer and NaCl. Proteinase K was added, and the mixture was incubated for one hour at 55 °C and then inactivated at 95 °C. The samples were extracted with phenol-chloroform–isoamyl alcohol 3-4 times and centrifuged. DNA was isolated using Anhydrous ethanol and 70% cold ethanol. After the ethanol evaporated, the DNA was suspended in TE buffer and mixed with RNase for 1 h. DNA concentrations were measured using a NanoDrop Instrument (ThermoScientific, Willmington, DE, USA). All of the DNA samples were stored at −20 °C before further analysis.

**PCR and Pyrosequencing**

Oligonucleotide primers targeting the V3-V5 hypervariable regions of 16S rRNA were designed based on bacterial universal primers. The regions were integrally amplified by PCR using fusion primers composed of 454 FLX Titanium sequencing primers (primer A, 5ʹ-CCATCTCATCCCTGCGTGTCTC CGACTCAG-3ʹ and primer B, 5ʹ-CCTATCCCCTGTGTGCCTTGGCAG TCTCAG-3ʹ), a unique 10 nt barcode, and V3-V5 primers (517F, 5'-ACTCCTACGGGAGGCAGCAG-3' and 907 R, 5ʹ-CCGTCAATTCM TTTGAGTTT-3ʹ). The PCR protocol included an initial denaturation at 95 °C for 2 min; 30 cycles that consisted of denaturation at 95 °C for 20 s, annealing at 50 °C for 30 s, and elongation at 72 °C for 5 min; and a final extension at 72 °C for 5 min. The PCR products were purified using 1.0X AMPure XP beads. Emulsion-based amplification and sequencing were performed according to the 454 FLX manual.

**Sequencing Data Analysis**

The unique reads were obtained through by processing the raw sequencing data using Mothur (v1.31.2, [http://www.mothur.org/)[1]](http://www.mothur.org/)(Schloss) and de-noised using the PyroNoise algorithm[2]. To facilitate the analysis of species diversity, the unique reads were clustered into operational taxonomic units (OTUs) using Mothur with the average neighbor clustering algorithm. The Ribosomal Database Project Classifier program (16S rRNA training set 9, [http://www.mothur.org/wiki/RDP_reference_files)[3]](http://www.mothur.org/wiki/RDP_reference_files)(Quince) was used for the taxonomy assignment of OTUs with an 80% bootstrap score. Subsequently, the sequences were aligned, profiling charts and tables were generated, Venn[4] diagrams were drawn, and phylogenetic trees were built.

Rarefaction curves and alpha diversity estimates were calculated by using Mothur to determine the species richness, observed species, ACE, Chao index, phylogenetic diversity, Shannon Weaver index, and Simpson index[5]. Community comparative analyses were performed using the web-based service UniFrac[6], and principal component analyses were performed using the SPSS Data Analysis Program version 12.0 (SPSS Inc, Chicago, IL, USA). The neighbor joining tree was constructed using the MEGA 4.0 program based on the Jukes-Cantor model.

Beta diversities based on UniFrac metrics were computed by QIIME programs (v1.50)[7]. Heatmaps of the microbial taxa were generated by MultiExperiment Viewer software[8](version 4.8.01; [http://www.tm4.org](http://mail.qq.com/cgi-bin/mail_spam?action=check_link&spam=0&spam_src=1&mailid=ZL1104-RtilPelwbbinIx5LeM_PR54&url=http://www.tm4.org)). The differences between sample pairs were determined from the neighbor joining tree by using the weighted UniFrac metrics.

**Statistical Analysis**

Summary and descriptive statistics (mean, median, standard error, range, and 95% confidence intervals) were generated for all samples. Bacterial loads were transformed using the natural logarithm to make boxplots. The cutoff value for assigning a sequence to a species-level phylotype was ≥97% similarity. Statistical significance tests were performed using the R program. The differences in the diversity indexes or bacterial community patterns among H1, H2, C2 groups were evaluated using the Wilcoxon rank-sum analysis or Kruskal-Wallis analysis of the variance (ANOVA) test, and were corrected by Benjamini-CHochberg[9]. The software Metastats (http://metastats.cbcb.umd.edu/) are used to analysis the significant difference between groups of samples. The 'p.adjust' in software R(v3.0.3) is used to calibrate the p value, and the calibration method is 'Benjamini-CHochberg'. The prevalences of the taxa were compared using Fisher’s exact test. Principal coordinates analyses and the unweighted pair group method with arithmetic mean (UPGMA) were performed using the weighted Fast UniFrac web interface. Unless otherwise noted, significance was considered at an alpha value of 0.05.

**References**

1. Schloss PD, Westcott SL, Ryabin T, Hall JR, Hartmann M, Hollister EB, Lesniewski RA, Oakley BB, Parks DH, Robinson CJ, et al. 2009. **Introducing mothur: open-source, platform- independent, community-supported software for describing and comparing microbial communities.** *Applied and environmental microbiology.* 75: 7537-7541.

2. Quince C, Lanzen A, Davenport RJ and Turnbaugh PJ. 2011. **Removing noise from pyrosequenced amplicons**. *BMC bioinformaticsx.* 12: 38.

3. Wang Q, Garrity GM, Tiedje JM, Cole JR. 2007. **Naive Bayesian classifier for rapid assignment of rRNA sequences into the new bacterial taxonomy**. *Appl Environ Microbiol.* 73:5261–5267.

4. Hanbo C. & Paul C.B. 2011. **VennDiagram: a package for the generation of highly-customizable Venn and Euler diagrams in R**. *BMC Bioinformatics.* 12:1-7.

5. Crawford P. A, Crowley J. R, Sambandam N, Muegge B. D, Costello E. K, Hamady M, Knight R, Gordon JI. 2009. **Regulation of myocardial ketone body metabolism by the gut microbiota during nutrient deprivation.** *Proc Natl Acad Sci USA.* 106(27), 11276-11281.

6. Lozupone C, Hamady M, Knight R. 2006. **UniFrac—an online tool for comparing microbial community diversity in a phylogenetic context**. *BMC Bioinform.* 7:371.

7. Caporaso JG, Kuczynski J, Stombaugh J, Bittinger K, Bushman FD, Costello EK, Fierer N, Pena AG, Goodrich JK, Gordon JI, et al. 2010. **QIIME allows analysis of high-throughput community sequencing data.** *Nature methods.* 7: 335-336.

8. Saeed A, Sharov V, White J, Li J, Liang W, Bhagabati N, Braisted J, Klapa M, Currier T, Thiagaraian M, et al. 2003. **TM4: a free, opensource system for microarray data management and analysis.** *Biotechniques.* 34:374.

9. James RW, Niranjan N, Mihai P. **Statistical Methods for Detecting Differentially Abundant Features in Clinical Metagenomic Samples**. PLoS computational biology. 2009
