# Supplementary material for: Pyrosequencing of Plaque Microflora In Twin Children with Discordant Caries Phenotypes
Source: PLoS One. 2015 Nov 2;10(11):e0141310. doi: 10.1371/journal.pone.0141310 (PMC4629883; doi:10.1371/journal.pone.0141310)
Supplement: S1 Table — Daily habits including caregivers, life habits, oral health awareness and oral status of parents and caregivers.Oral health habits including feeding within three months, brushing start time, brushing frequency and time ofday, brushing assistance.Eating habits including dietary preferences, poor eating habits, mainly concern on caries-related dietary factors. (DOC) [file pone.0141310.s004.doc]

**Table S1. Summary of questionnaire.**

| Kindergarten | Sample name | Zygosity determination | Sex | Daily habits^a^ | Oral health habits^b^ | Eating habits^c^ |
| --- | --- | --- | --- | --- | --- | --- |
| same kindergarten K1 | T1 | dizygotic (DZ) | opposite-sex | same | same | same |
|  | T2 |  |  |  |  |  |
|  | T3 |  |  |  |  |  |
|  |  |  |  |  |  |  |
|  | T4 | dizygotic (DZ) | opposite-sex | same | same | different |
|  | T5 |  |  |  |  |  |
|  | T6 |  |  |  |  |  |
|  | | | | | | |
| same kindergarten K2 | T7 | dizygotic (DZ) | opposite-sex | same | same | same |
|  | T8 |  |  |  |  |  |
|  | T9 |  |  |  |  |  |
|  |  |  |  |  |  |  |
|  | T10 | dizygotic (DZ) | opposite-sex | same | same | same |
|  | T11 |  |  |  |  |  |
|  | T12 |  |  |  |  |  |
|  | | | | | | |
| same kindergarten(a boarding school) K3 | T13 | dizygotic (DZ) | same-sex | same | same | different |
|  | T14 |  |  |  |  |  |
|  | T15 |  |  |  |  |  |
|  |  |  |  |  |  |  |
|  | T16 | dizygotic (DZ) | same-sex | same | same | same |
|  | T17 |  |  |  |  |  |
|  | T18 |  |  |  |  |  |
|  |  |  |  |  |  |  |
|  | T19 | dizygotic (DZ) | same-sex | same | same | different |
|  | T20 |  |  |  |  |  |
|  | T21 |  |  |  |  |  |
|  |  |  |  |  |  |  |
| same kindergarten K4 | T22 | dizygotic (DZ) | same-sex | same | same | same |
|  | T23 |  |  |  |  |  |
|  | T24 |  |  |  |  |  |
|  |  |  |  |  |  |  |
|  | T25 | unknow | same-sex | same | same | same |
|  | T26 |  |  |  |  |  |
|  | T27 |  |  |  |  |  |
|  |  |  |  |  |  |  |
|  | T28 | unknow | same-sex | same | same | same |
|  | T29 |  |  |  |  |  |
|  | T30 |  |  |  |  |  |

a Daily habits including caregivers, life habits, oral health awareness and oral status of parents and caregivers.

b Oral health habits including feeding within three months, brushing start time, brushing frequency and time of day, brushing assistance.

c Eating habits including dietary preferences, poor eating habits, mainly concern on caries-related dietary factors.
